# West Nile Virus Infection in Commercial Waterfowl Operation, Wisconsin

**DOI:** 10.3201/eid1209.051648

**Published:** 2006-09

**Authors:** Jennifer K. Meece, Tamara A. Kronenwetter-Koepel, Mary F. Vandermause, Kurt D. Reed

**Affiliations:** * Marshfield Clinic Research Foundation, Marshfield, Wisconsin, USA

**Keywords:** Agricultural workers, animal husbandry, ducks, geese, outbreak, universal precautions, West Nile virus, zoonoses, dispatch

## Abstract

A West Nile virus (WNV) outbreak occurred at a commercial waterfowl operation in Wisconsin in 2005. Retrospective analysis of dead and live birds was conducted. WNV was detected by PCR in 84.1% of 88 dead birds; neutralizing antibodies were found in 14 of 30 randomly sampled asymptomatic or recovered birds.

West Nile virus (WNV) is a zoonotic pathogen that cycles naturally between wild birds and mosquitoes. Although hundreds of avian species are susceptible to infection, few instances of disease in commercial flocks of domestic or exotic birds have been reported. WNV infection in domestic geese has been documented in Israel (*1*), Canada (*2*), and Hungary (*3*). These outbreaks were characterized by substantial deaths and high seroprevalence rates. In the United States, an outbreak of WNV associated with high seroprevalence but low death rates was documented in a commercial breeder turkey operation in Wisconsin (*4*). Nonvector transmission was hypothesized to have contributed to the intensity of these outbreaks. We report an outbreak of WNV in a commercial flock of exotic and domestic ducks and geese in Wisconsin in 2005 that was associated with substantial numbers of deaths and a high seroprevalence rate. WNV in this agricultural setting may also have been enhanced by nonvector routes.

## The Study

On August 8, 2005, Marshfield Laboratories (Marshfield, WI, USA) was contacted to test 2 deceased captive waterfowl from a farm with a suspected outbreak of WNV. Kidney, spleen, and oral and cloacal swabs were taken and tested by using reverse transcriptase (RT)-PCR (*5*). When tissues and swabs were found positive, we contacted the farm operator to determine the extent and nature of the outbreak and obtained permission to conduct a site visit. Two site visits were made. On the first, on August 18, 2005, we collected frozen, dead carcasses for testing and collected swabs, serum, or both from 8 clinically ill birds. At the second visit, on August 24–25, we collected serum samples.

The farm was primarily operated for production of breeding stock and included >25 species of domestic and exotic species of geese, ducks, and poultry. In a typical year of operation, ≈150 breeding stock are on the farm in early spring; by June the flock expands to ≈1,250 birds. Hatch-year birds are raised to adults and sold to breeders in the fall. An average of 3 deaths per month from various causes, including trauma and infections, occur in the flock. The birds are housed in large, clean, well-drained outdoor pens constructed of wood beams and large-gauge nylon netting. Birds are not segregated by species, and <200 birds may be housed together in individual pens. Mosquitoes and small wild birds can move easily through the netting. Each pen contains a concrete pond with continually circulated water that serves as a water source and resting area where the birds congregate each night.

Prior evidence of WNV had been documented on the farm in 2002 but was limited to 5 ducks that died shortly after weakness, tremors, and other neurologic signs developed. At necropsy, WNV was isolated from spleen, kidney, oral swabs, and cloacal swabs from these birds. Infection control interventions used at that time included draining and bleach sterilization of the concrete ponds (J.K. Meece and K.D. Reed, unpub. data).

The farm operator reported that on June 20, 2005, a single Ross goose was noted to have weakness, tremors, head tilt, and drooping wings. This bird died within a day of onset of signs. In the next 2 days, similar neurologic signs developed in 4 more Ross geese, and they died. For several weeks no additional deaths were observed, but from July 22 to July 30, six Siberian red-breasted geese and 2 American widgeons died after displaying neurologic signs. Thereafter, an average of 20 birds of various species died per week until August 17, when the outbreak abruptly ended. During the outbreak, the operator salvaged the birds and stored them at –20°C. Our first visit to the farm occurred on August 18, a day after the last dead bird was collected.

Dead birds (n = 88), saved frozen at –20°C, were returned to the laboratory for testing. The condition of the birds was highly variable; many of the birds had been pecked and partially cannibalized by other flock members. The farm operator identified the American widgeon, Eurasion widgeon, blue-winged teal, and green-winged teal as hatch-year birds. The other species were mixed ages. RNA was extracted from oral-pharyngeal swabs with the RNeasy mini protocol Qiagen kit (Qiagen, Inc., Valencia, CA, USA) and tested for WNV-specific RNA with real-time PCR with the Roche Light Cycler (Roche, Indianapolis, IN, USA) (*5*). Birds that tested negative by oral swabs were necropsied, and their kidney tissues were tested with the same protocol. Overall, 74 (84.1%) of 88 of the dead birds tested were positive for WNV. To assess the sensitivity and specificity of the PCR, diluent from oral swabs from 5 WNV-positive birds and 5 WNV-negative birds were injected onto African green monkey kidney cells (Vero cells, American Type Cell Culture #81-CCL, Manassas, VA, USA) for virus isolation. WNV was recovered in culture from all birds that were PCR positive but not from those that were PCR negative (100% concordance). These culture results were also confirmed by RT-PCR (Table).

**Table:** Species of dead birds collected at investigation farm*

Species of dead birds	WNV positive, n (%)	No. WNV negative
Blue-winged teal (*Anas discors*)	16 (100)	0
Northern pintail (*A. acuta*)	4 (100)	0
Green-winged teal (*A. carolinensis*)	3 (100)	0
Falcated teal (*A. falcate*)	3 (100)	0
Northern shoveler (*A. clypeata*)	1 (100)	0
Barrow's goldeneye (*Bucephala islandica*)	1 (100)	0
Cackling goose (*Branta hutchinsii*)	1 (100)	0
Eurasian widgeon (*A. penelope*)†	25 (96.1)	1
American widgeon (*A. americana*)	15 (93.8)	1
Siberian red-breasted goose (*Branta ruficollis*)	3 (50.0)	3
Ross goose (*Chen rossii*)	2 (40.0)	3
Mountain quail (*Oreortyx pictus*)	0	2
Common eider *(Somateria mollissima)*	0	1
Hooded merganser (*Lophodytes cucullatus*)†	0	1
Canvasback (*Aythya valisineria*)†	0	1
Domestic turkey (*Melleagris gallopavo*)	0	1

*WNV, West Nile virus. †Species reported to Centers for Disease Control and Prevention avian mortality database as having tested positive for WNV from 1999 to present (*6*).

Eight live birds that the owner identified as having displayed neurologic signs were captured for sample collection (1 Siberian red-breasted goose, 1 Barrow's goldeneye, 1 blue-winged teal, 2 Eurasian widgeons, 2 Ross geese, and 1 wood duck). Swab samples were obtained from oral and cloacal cavities of these birds. Serum samples, collected from 3 of these birds (1 Siberian red-breasted goose, 1 Barrow's goldeneye, and1 blue-winged teal), showed high antibody titers to WNV. These data are included in the serologic results for cohort B (see below). We detected virus from the oral cavity of 1 of the live, clinically ill Eurasian widgeons.

To assess the extent of WNV exposure to the flock, serum samples from 2 mixed-age cohorts were collected to test for specific antibodies to WNV and Saint Louis encephalitis virus (SLEV) by a constant virus serum dilution neutralization assay during the second site visit (*7*). Cohort A was a group of 58 geese (45 Ross geese, 7 snow geese, and 6 blue geese) that had been removed from the farm at the first sign of bird death and relocated to a site 15 miles away. Cohort B was a group of 12 ducks and 18 geese (9 Siberian red-breasted geese, 2 bar-headed geese, 6 Ross geese, 1 blue goose, 6 Eurasian widgeons, 1 wood duck, 1 redhead duck, 1 Barrow's goldeneye, 2 blue-winged teals, and 1 northern shoveler) housed continuously at the outbreak site; these birds were from the same 3 pens where the bird deaths occurred. WNV-specific antibodies were detected in serum from 5 (8.6%) of 58 birds in cohort A. No antibodies to SLEV were detected in cohort A. All 5 of the seropositive birds were Ross geese; 1 of the 5 was identified as a 2005 hatch-year bird. Antibody titers for cohort A ranged from 10 to 80. WNV-specific antibodies were detected from 14 (46.7%) of 30 birds in cohort B. No antibodies for SLEV were detected in cohort B. Excluding the 3 clinically ill birds (titers below), positive antibody titers were detected in the Siberian red-breasted goose (n = 2), bar-headed goose (n = 1), Eurasian widgeon (n = 5), blue goose (n = 1), Ross goose (n = 1), and blue-winged teal (n = 1). Cohort B was a mixed-age cohort, and we did not determine the age of individual birds in this sampling group. Antibody titers for cohort B ranged from 10 to >320. The farm owner identified 3 birds in this cohort as having been clinically ill. These birds were a Siberian red-breasted goose, a Barrow's goldeneye, and a blue-winged teal with titers of 160, >320, and 160, respectively.

At the onset of the investigation, infections due to avian influenza and exotic Newcastle disease virus were considered in the differential diagnosis. Oral swabs from all dead birds were tested at the Wisconsin Veterinary Diagnostic Laboratory (Madison, WI, USA) for both agents and were negative.

## Conclusions

This report is the first to document WNV in a commercial waterfowl operation in the United States. The extent of this outbreak, as evidenced by the seroconversion rate in cohort B, far exceeded deaths in the flock. This outbreak caused a considerable economic loss for the operator, and the occurrence of infection among a large number of birds posed a major occupational hazard to the farm workers.

Our study has limitations because it was a retrospective analysis and we were not able to collect some key data, such as vector infection rates, or to sample the water for WNV. However, the concentrated loss of birds within a small number of housing pens during late July and early August, along with the high seroconversion rate among asymptomatic and recovered birds in the flock, suggests that nonvector transmission may have occurred. Certain behavior traits of waterfowl may facilitate this phenomenon. Sick birds are regularly pecked and cannibalized by other members of the flock. Previous studies have documented that feather pulp in infected birds often contains high titers of WNV (*8*). In addition, the tendency of waterfowl to congregate on ponds at night provides an opportunity for nonvector transmission through prolonged contact with virus shed into a common water source (*9*). During the mid to late summer, when WNV transmission is highest, most birds in this commercial flock were hatch-year birds and may have been more susceptible to infection (*2*). In contrast, older birds that may have immunity due to prior exposure to WNV made up the minority of the bird population.

Because avian influenza and exotic Newcastle disease were the only other pathogens tested for in this outbreak, we cannot completely rule out the possibility that coinfection with other pathogens contributed to death in certain species. Previous studies have shown that coinfection and other stressors can contribute to high death rates within captive flocks (*3*).

In this outbreak we saw no evidence of symptomatic infection with WNV among the limited number of workers regularly exposed to the birds. In contrast, the outbreak of WNV among breeder turkeys in Wisconsin in 2002 was heralded by illness among farm workers (*4*). In silent outbreaks, the potential risk for humans is masked yet may still be substantial (*10*). In light of growing concerns about a possible avian influenza pandemic, universal precautions, as outlined by the US Department of Labor Occupational Safety and Health Administration (*11*), should be applied when working in avian husbandry. Additionally, more timely reporting of suspected outbreaks to public health officials would permit comprehensive investigations that could elucidate the transmission dynamics of disease in agricultural settings. Timely reporting is also important in implementing control strategies that mitigate spread of infectious diseases to farm workers.
